# Demography and Population Projection of *Tetranychus urticae* (Tetranychidae) on *Phaseolus vulgaris* (Fabaceae) Colonized by Entomopathogenic Fungal Endophytes

**DOI:** 10.3390/insects15010073

**Published:** 2024-01-21

**Authors:** Pengxiang Hong, Chandra Kanta Dash, Muhammad Adeel Ghafar, Inzamam Ul Haq, Liuyang Lu, Chenghua Zhou, Qing Wu, Liande Wang

**Affiliations:** State Key Laboratory of Ecological Pest Control for Fujian and Taiwan Crops, Key Laboratory of Biopesticide and Chemical Biology, MOE, College of Plant Protection, Fujian Agriculture and Forestry University, Fuzhou 350002, Chinadashck.entom@sau.ac.bd (C.K.D.); adeelbhatti069@gmail.com (M.A.G.);

**Keywords:** *Tetranychus urticae*, two-sex life table, *Beauveria bassiana*, *Phaseolus vulgaris*, population projection

## Abstract

**Simple Summary:**

The two-spotted spider mite *Tetranychus urticae* and its interactions with fungi, notably *Beauveria bassiana*, on common bean plants were the main subjects of this study. The mite’s life cycle on untreated and endophyte-colonized plants was examined using the age-stage, two-sex life table hypothesis. The findings indicated that mites reared on untreated plants had shorter developmental stages and higher fecundity than those on colonized plants. Furthermore, mites on untreated plants displayed a higher intrinsic rate of increase and net reproductive rate. The study also revealed that fungal endophyte colonization negatively affected mite growth, adult lifespan, fecundity, reproductive rate, and the intrinsic rate of increase. The findings point to a possible application of entomopathogenic fungal endophytes in host plant resistance programs for future integrated pest control.

**Abstract:**

*Tetranychus urticae* is a highly polyphagous and global pest. Spider mites primarily feed on the underside of leaves, resulting in decreased photosynthesis, nutritional loss, and the development of chlorotic patches. We investigated the life tables of the two-spotted spider mite *T. urticae* on fungal endophyte *Beauveria bassiana* colonized and untreated plants of the common *Phaseolus vulgaris* L., a bean plant. Based on the age-stage, two-sex life table theory, data were evaluated. The mites raised on untreated plants had protonymphs, deutonymphs, and total pre-adult stage durations that were considerably shorter (1.76, 2.14, and 9.77 d, respectively) than the mites raised on plants that had been colonized (2.02, 2.45, and 10.49 d, respectively). The fecundity (F) varied from 28.01 eggs per female of colonized plants to 57.67 eggs per female of endophyte-untreated plants. The net reproductive rate (*R*_0_) in the plants with and without endophytes was 19.26 and 42.53 brood, respectively. The untreated plants had an intrinsic rate of increase (*rm*) of 0.245 days as opposed to the colonized plants, which had an *r* of 0.196 days and a finite rate of increase (*λ*) (1.27 and 1.21, respectively). Population forecasts based on a two-sex, age-stage life table demonstrated the dynamism and variability of the stage structure. Furthermore, the colonization of *B. bassiana* had a negative impact on the growth and development of *T. urticae*. It lowered the adult mite life span, female fecundity, net reproduction rate, and intrinsic growth rate. We propose that future research should better use entomopathogenic fungal endophytes to understand host plant resistance strategies in integrated pest management.

## 1. Introduction

*Tetranychus urticae* Koch (Acari: Tetranychidae), a two-spotted spider mite, infests more than 1110 plant species from 250 different plant families, making it a highly polyphagous and global pest [1,2,3]. Spider mites primarily feed on the underside of leaves. They do this using their stylet to pierce the leaf tissues and scavenge the cell contents. This results in cell collapse owing to decreased photosynthesis, nutritional loss, and the development of chlorotic patches. A serious infestation may result in browning, defoliation, and even premature wilting and the death of plants. The main approach for controlling pest mites is chemical insecticides, which are still used, and acaricides. However, within a few years of the introduction of any novel substance, spider mites can become resistant to specific chemicals [2]. Because of the widespread application of insecticides and the biological traits of the mites, such as their high reproductive capacity and arrhenotokous type of reproduction, they are exceedingly challenging to control. Thus, researchers are looking for alternate biological control strategies to manage this pest, including predatory mites [4] and entomopathogenic fungi [5,6].

Predatory mites, particularly phytoseiid mites like *Phytoseiulus persimilis* Athias-Henriot, have been employed in greenhouses for an extended period to control spider mites. The use of fungal entomopathogen is also increasing, and several Hypocreales-based mycopesticides from different entomopathogenic fungi are now available commercially [7]. The efficiency of entomopathogenic fungi in laboratories, greenhouses, and field conditions has been reported [5,6,7,8]. However, the above two options, predatory mites or entomopathogenic fungi, cannot provide optimum pest control. Their application in combination is also not always compatible.

Entomopathogenic fungi, traditionally known for their pathogenicity against insect pests, have recently been shown to play multiple roles besides their entomopathogenic characteristics, like rhizosphere colonizer [8,9], plant growth promoter [10,11], plant disease antagonist [12,13], and particularly endophytic establishment in plants [14,15]. The latter is especially promising because several studies reported that entomopathogenic fungi like *Beauveria*, *Metarhizium*, *and Lecanicillium* could colonize plants upon artificial inoculation, and their planta establishment can provide several benefits to the host plant, for example, protection from herbivores and plant disease [16]. A previous study reported that *B. bassiana* could be colonized endophytically after seed inoculation. In comparison to endophyte-free plants, the development and reproduction of two-spotted spider mites were significantly reduced in the bean plants colonized by the endophyte [17].

Selecting resistant plants is a first and vital component of pest management. An increasing number of studies demonstrate that colonization by endophytic entomopathogenic fungi makes host plants unsuitable for insect pests by negatively affecting their growth and reproduction when insects feed on the colonized plant [18]. However, to date, no study reports the detailed life table of pest insects in endophyte-colonized plants. Growth and development with the reproductive data on a particular host provide valuable information on the population ecology. The life table includes all the population parameters, such as the intrinsic rate of increase, finite rate of increase, net reproductive rate, age-stage differentiation, biography parameters, comparative survival rate, and reproduction rate. Every life table is a useful tool for assessing a population’s fitness. Data from life tables can also be used to anticipate damage, estimate population growth, and determine when to apply pest control measures [19]. The objectives of this study are to investigate the demography of *T. urticae* on *Phaseolus vulgaris* L (Fabaceae) plants colonized by entomopathogenic fungal endophytes and to project the population dynamics of the mites under the influence of these endophytes. Specifically, the study aims to elucidate how the presence of entomopathogenic fungal endophytes in the host plants affects the life stages, survival, and reproductive parameters of *T. urticae*, providing insights into potential implications for pest management strategies.

## 2. Materials and Methods

### 2.1. Fungal Isolate Preparation and Preparation of Conidia

The *Bassiana strain* (B16) used in this study was obtained from the Fuzhou, Fujian, Fujian Agriculture and Forestry University (FAFU) campus, Fuzhou, China. Using Humber’s key [20], the strain was recognized morphologically. The molecular approach (DNA sequence was acquired from the ITS region or nuclear rDNA(s), Gene Bank accession number MG844431) was used to supplement the identification. In 9 cm plastic Petri plates, the fungus was grown on yeast extract SDAY (supplemented potato dextrose agar) and cultured in complete darkness for 10–14 days at 25 ± 1 °C and 60–70% RH. Conidia were collected by scraping the surface of the sporulating PDAY plate into an aqueous solution containing distilled sterile water and 0.01% Tween 80 using a sterile scalpel. The suspension was vortexed for two minutes to separate the conidia from the hyphal debris. After that, the suspension was run through numerous layers of cheesecloth. Germany’s upgraded Neubauer hemocytometer regulated the spore concentration to 1 × 10^8^ spores/mL.

The viability of the conidia was determined before inoculation by spread-plating 0.1 mL of a 1 × 10^4^ conidia mL^−1^ suspension on the PDA plates. For 24 h, the plates were incubated at 25 ± 1 °C. Using an Olympus CX21FS1 light microscope (Olympus Corporation, Tokyo, Japan), 100 spores were counted under the microscope to measure the percentage of conidial germination. Four replications of each plate were made. In all of the experiments, the conidial germination exceeded 95%.

### 2.2. Seed Inoculation with Entomopathogenic Fungi

Bean seeds were treated with 70% ethyl alcohol for two minutes, 1.5% sodium hypochlorite (NaOCl) for three minutes, and then three times with sterile distilled water to surface-sterilize them. To evaluate the efficacy of the surface sterilization method, the third wash was applied to PDA (potato dextrose agar) media along with breeding at 25 °C [17]. After surface sterilization, the seeds were treated (conidial suspension) for 16 h or left untreated (sterile distilled water with 0.1% Tween 80). Then, the seeds that had been sterilized were put into plastic pots (9 cm × 7 cm) with a planting substrate (compost and vermiculite). Before being put into pots, the planting substrate was autoclave sterilized at 121 °C for two hours. After sowing three seeds in each pot, one healthy plant was retained and the other two were removed by hand picking upon germination. 

### 2.3. Evaluation of Plant P. vulgaris Endophytic Colonization

One week following seed inoculation with *B. bassiana*, the evaluation of *P. vulgaris* endophytic colonization was conducted through a series of steps. First, plant roots were carefully excavated, and adhering soil was gently removed. Subsequently, the roots were surface sterilized using a 70% ethanol solution, followed by a 10% sodium hypochlorite solution, each for one minute. The roots were sectioned into segments after thorough rinsing with sterile distilled water. These segments were then placed onto potato dextrose agar (PDA) plates to assess the presence and extent of *B. bassiana* colonization. Fungal structures such as hyphae and conidia were microscopically observed, and the degree of colonization was quantified based on the frequency and density of fungal structures within the root tissues.

### 2.4. Spider Mites

The spider mite rearing and colony maintenance were conducted using an initial population of *T. urticae* obtained from the State Key Laboratory of Ecological Pest Control for Fujian and Taiwan Crops, College of Plant Protection, Fujian Agriculture and Forestry University, Fuzhou, China. The initial population of *T. urticae* was reared on bean plants sown in pots in the lab. The mites were introduced onto host plants in a controlled environment with optimal conditions for plant growth, including regulated temperature (20–30 °C) and humidity (40–60%). The population was regularly monitored through visual inspections and counting methods to assess signs of activity and population growth. Host plants were consistently provided to sustain the mites’ feeding and nutritional needs. Colonies were isolated to prevent contamination, and the meticulous removal of dead or diseased individuals was carried out. Generation transitions were initiated by allowing mated females to lay eggs, and the periodic introduction of individuals from the source population ensured genetic diversity. Harvesting for experimental purposes involved gently brushing or tapping mites into containers.

### 2.5. Life Table Study

**Figure 1:**
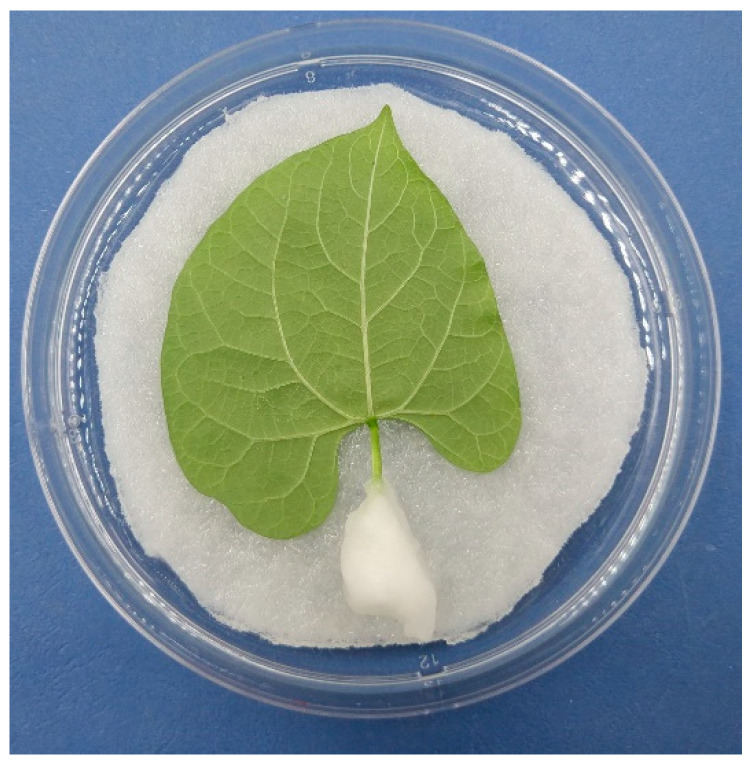
Experimental unit for life table study of *T. urticae* (©Author).

In our study, we utilized eighty primary leaves from both inoculated and control bean plants. Each leaf was placed upside down on a filter cotton mat in a Petri dish, with the petiole wrapped in cotton wool to prevent desiccation (Figure 1). On each leaf, a *T. urticae* female, aged 3–5 days, was introduced. The purpose of these females was solely to obtain uniformly aged eggs; they were not used in the life table analysis. We meticulously counted the eggs laid within a 24 h period under a microscope, ensuring a consistent age for all eggs used in the study. After collecting these eggs, we selected 50 per treatment, across three replicates, for our life table analysis. The environmental conditions were consistently maintained, and we performed regular observations to track the development of the mites, noting the duration of each life stage, mortality instances, and stage transitions. The data thus collected were instrumental in constructing an accurate life table, allowing us to calculate life expectancy and other crucial demographic parameters.

### 2.6. Population Projection

Data obtained from the age-stage, two-sex life table on development, survival, and fecundity were used to calculate the population growth of *T. urticae* on the endophyte-colonized and untreated host plants based on the method of Chi [21]. Projections were generated by the computer software TIMING-MSChart [22].

### 2.7. Statistical Analyses

The data of *T. urticae* on the endophyte-colonized and untreated host plants were collected and analyzed according to the age-stage, two-sex life table theory [23] and the methodology given by Chi and Liu [22] using the TWO SEX-MSChart software [24]. *S_xj_* (the survival rate of *T. urticae* from egg to *x* days and stage *j*), *l_x_* (the specific survival rate of *T. urticae* with respect to age), *f_xj_* (the fecundity of *T. urticae* with respect to a specific age and stage), *m_x_* (the fecundity of *T. urticae* with respect to a specific age), *l_x_m_x_* (the net maternity of *T. urticae* with respect to a specific age), and the population parameters of *T. urticae* were determined, including the mean generation time (*T*), the net reproduction rate (*R*_0_), the intrinsic rate of increase (*r*), and the finite rate of increase (*λ*). These were calculated using the following equations:

The specific survival rate of *T. urticae* with respect to age: ($l_{x})=\overset{m}{\underset{j=1}{\sum }}s_{xj}$.

Here, m represents the number of stages of *T. urticae* in the experiment. 

The fecundity of *T. urticae* with respect to a specific age: $\left(m_{x}\right)=\frac{\sum_{j=1}^{m}s_{xj}f_{xj}}{\sum_{j=1}^{m}s_{sj}}$.

The intrinsic rate of increase: (*rm*) =$\overset{\infty }{\underset{x=0}{\sum }}e^{-r\left(x+1\right)}l_{x}m_{x}=1$.

The finite rate of increase: ($\lambda )=e^{r}$.

The net reproduction rate: ($R_{0})=\overset{\infty }{\underset{x=0}{\sum }}l_{x}m_{x}$.

The mean generation time: ($T)=\frac{lnR_{0}}{r}$.

The life expectancy with respect to a specific age: ($e_{xj})=\overset{\infty }{\underset{i=x}{\sum }}.\overset{m}{\underset{y=j}{\sum }}s{’}_{iy}$.

Here, *s′_iy_* is the probability that *T. urticae* of age *x* and stage *j* will survive to age (*i*) and stage (*y*). 

The reproductive value: ($v_{xj})=\frac{e^{r\left(x+1\right)}}{s_{xj}}\overset{\infty }{\underset{i=x}{\sum }}e^{-r\left(i+1\right)}\overset{m}{\underset{y=i}{\sum }}s{’}_{iy}f_{iy}$.

The contribution of individuals of age *x* and stage *j* to the future population. 

In this experiment, bootstrap tests were performed to check differences across all the treatments at a 5% significance level based on the confidence interval of difference [25]. The matched bootstrap test was used to evaluate distinctions between treatments. The TWOSEX-MS Chart software included these steps in its code.

## 3. Results

### 3.1. Endophytic Colonization

**Figure 2:**
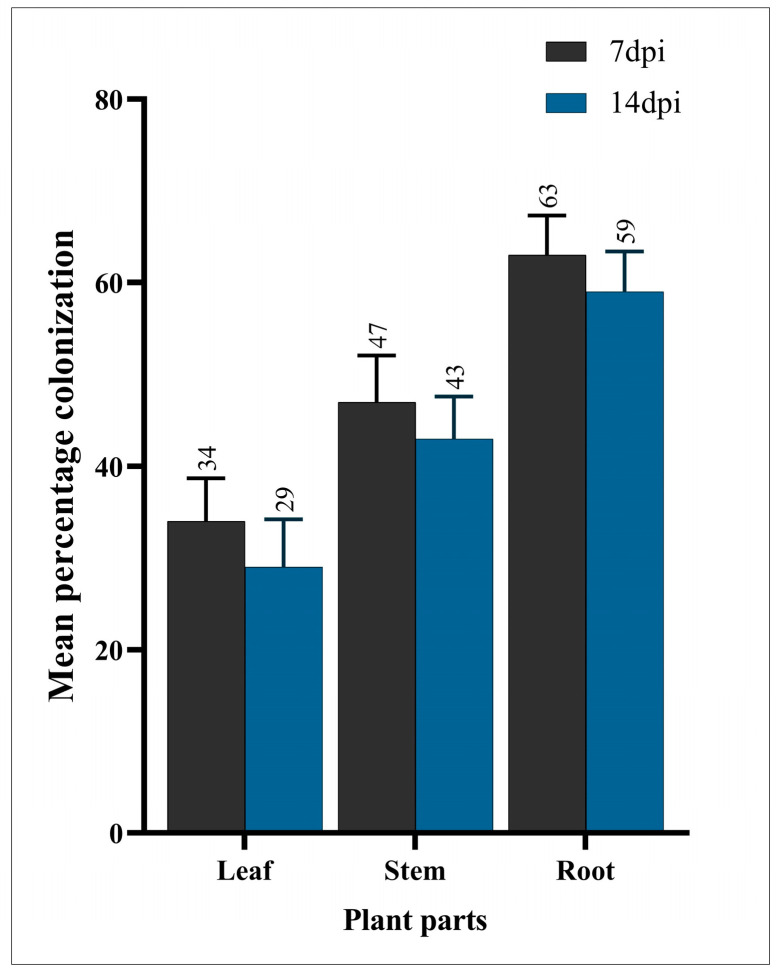
Mean percentage colonization of fungus in different parts of *P. vulgaris* 7 days after inoculation (7 dpi) and 14 days after inoculation (14 dpi).

In the evaluation of *P. vulgaris* endophytic colonization by *B. bassiana* following inoculation, the results indicated a successful establishment of the fungal endophyte within the plant leaves, stems, and roots. The microscopic examination revealed prominent hyphal structures and conidia distributed throughout the tissues, confirming the presence of *B. bassiana*. The quantitative analysis demonstrated a significant frequency of fungal structures in the plant parts, with a notable density of colonization observed in the vascular and cortical regions (Figure 2).

### 3.2. Key Life History Data of Tetranychus urticae

Table 1 outlines the developmental durations associated with each stage of the life cycle. The egg stage exhibited a range of 3.78 to 3.82 days, depending on the treatment applied. Concurrently, larval development spanned from 2.08 to 2.15 days. However, a marked distinction emerged when spider mites fed on the colonized plants, particularly in the protonymph and deutonymph stages, which lasted 2.02 and 2.45 days, respectively, compared to 1.76 and 2.14 days in the untreated plants. The cumulative pre-adult stage duration was 9.77 days for the untreated plants and extended to 10.49 days for the colonized plants. Significantly, the endophytic augmentation of bean vegetation by entomopathogenic *B. bassiana* substantially influenced adult longevity. On the untreated plants, the adult longevity averaged 12.88 days, whereas on the endophyte-colonized plants, it diminished significantly to 9.05 days. The results of the current experiment showed a significant effect on the adult pre-oviposition period (APOP), total oviposition period (TPOP), pre-adult survival rate, and oviposition days compared to control. A significantly shorter APOP (0.81 ± 0.001 days) and number of oviposition days (7.50 ± 0.19 days) were recorded in the colonized plants compared to the control (0.84 ± 0.001 days and 11.10 ± 0.23 days). Similarly, the total oviposition period (TPOP) was also significantly shorter on the colonized plants (10.76 ± 0.11 days) compared to the untreated plants (11.56 ± 0.13 days).

### 3.3. Demographic Parameters

Table 2 contains a list of demographic parameters, including the net reproduction rate (*R*_0_), mean generation time (*T*), intrinsic rate of rise (*rm*), and finite rate (*λ*). In comparison to endophyte-colonized plants (19.26), the endophyte-free plants (42.53) had a much greater net reproductive rate (*R*_0_). Similar to this, the endophyte-free plants showed considerably higher intrinsic rates of increase (*r*) (0.245 and 0.196, respectively) and finite rates of increase (*λ*) (1.27 and 1.21, respectively). The statistics show that the treated and untreated plants had similar mean generation times (*T*).

**Figure 3:**
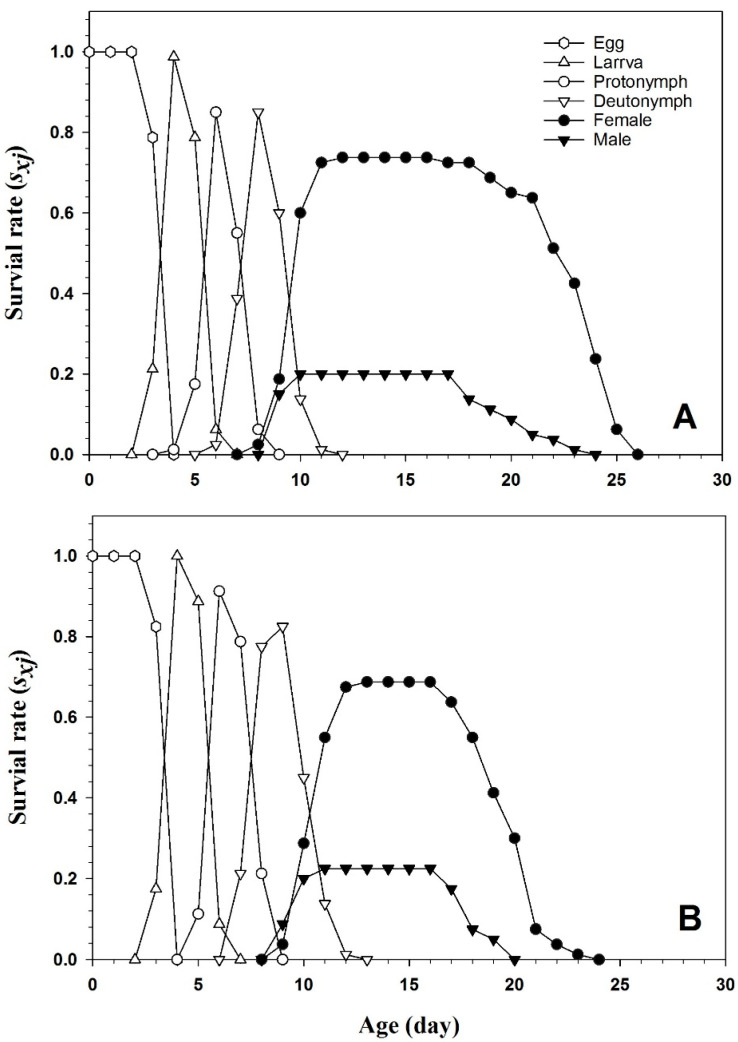
Age-stage-specific survival rate (*s_xj_*) of *T. urticae* on endophyte-free (**A**) and colonized (**B**) plants.

Figure 3 displays the survival rate curves (*s_xj_*) of *T. urticae* raised on the endophyte-colonized and endophyte-free plants. The age-stage-specific survival rate (*s_xj_*) represented the survival rate of *T. urticae* from an egg to age *x* (days) and stage *j*. Compared to Nm/N, the higher curves seen in the female adults suggest a higher proportion of Nf/N. The age-stage survival curve (*s_xj_*) revealed overlap between succeeding stages, indicating that the *T. urticae* individuals developed at different rates. The age-stage-specific survival rate of the females on the endophyte-colonized plants (0.72) was considerably lower compared to the females (0.76) on the endophyte-free plants.

**Figure 4:**
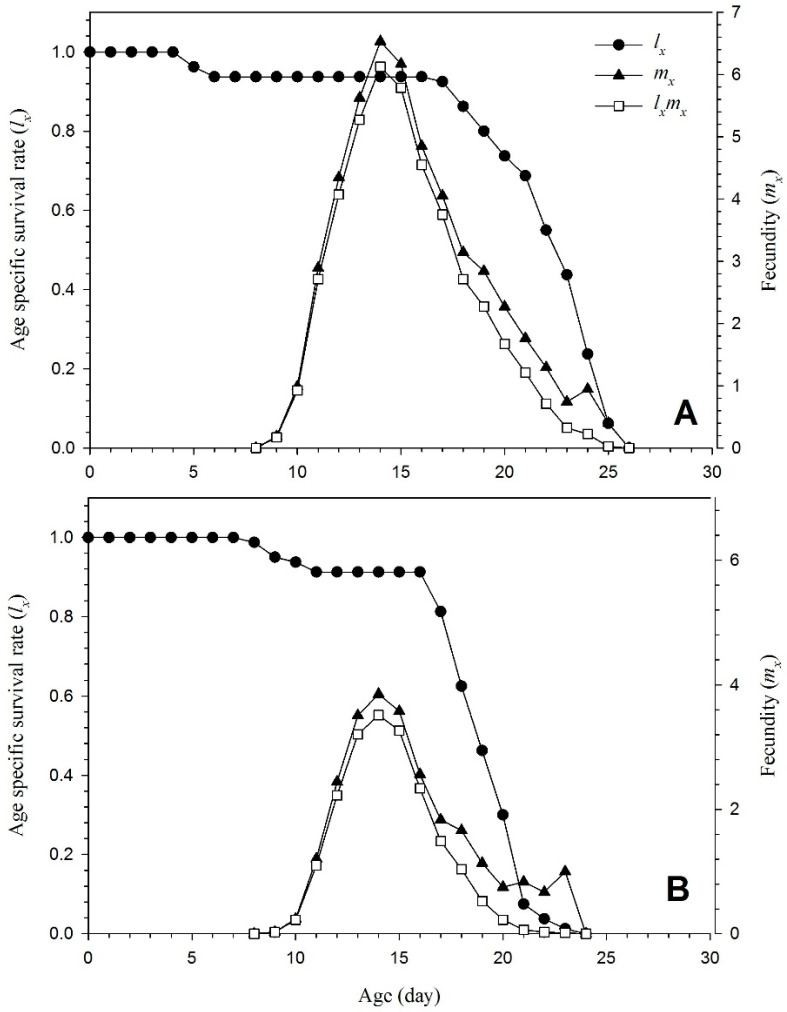
Age-specific mortality (*l_x_*), fecundity (*m_x_*), and maternity (*l_x_m_x_*) of *T. urticae* reared on endophyte-free (**A**) and colonized (**B**) plants.

Figure 4 displays the fecundity (*m_x_*), maternity (*l_x_m_x_*), and age-specific survival rate (*l_x_*) of *T. urticae* raised on plants both colonized by and devoid of endophytes. All the surviving individuals of each gender are combined with those who died during the pre-adult phases to create *l_x_*. It shows the survival rate of *T. urticae* from an egg to age *x* (days). The curves in Figure 4 are all condensed into the *l_x_* curve. The female age-specific fecundity (*m_x_*) revealed reduced fertility in the endophyte-colonized plant. Additionally, the daily fecundity (4 eggs) was considerably lower than in the untreated plants (6.7 eggs).

**Figure 5:**
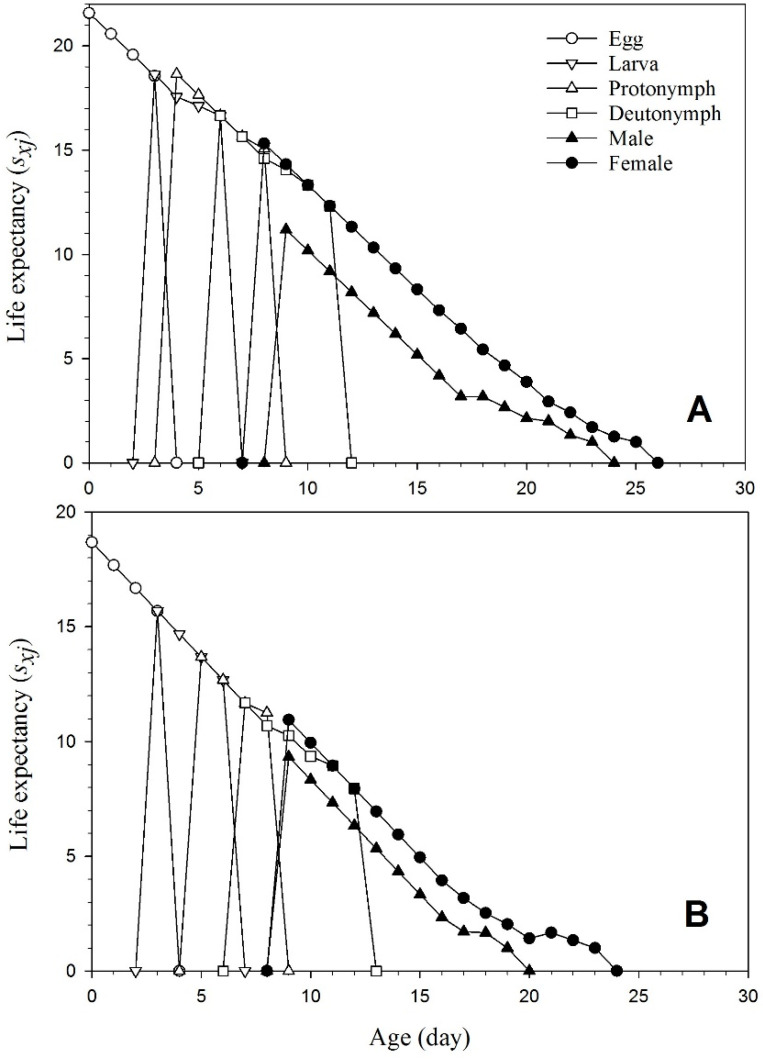
Age-stage-specific life expectancy (*e_xj_*) of *T. urticae* reared on endophyte-free (**A**) and colonized (**B**) plants.

Figure 5 illustrates an increase in life expectancy (*e_xj_*) all mites in both endophyte-colonized and endophyte-free plants. This indicates the anticipated time that a mite at stage *j* and age *x* will survive. A newborn egg’s life expectancy (*e*_01_) is the same as the average lifespan. In the mites raised on the endophyte-colonized plants, the lifespan of a newly placed egg was shorter.

**Figure 6:**
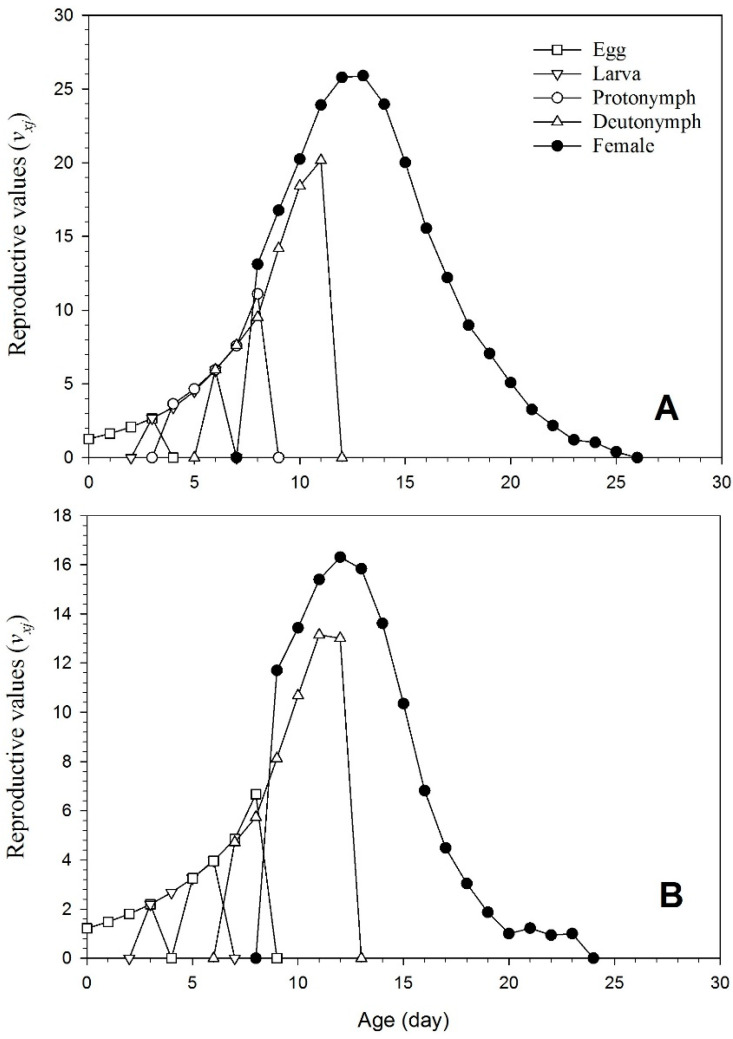
Reproductive values for each age stage of *T. urticae* raised on endophyte-free (**A**) and colonized (**B**) plants.

Figure 6 displays *v_xj_* (reproductive value), which is the contribution of individuals of age *x* and stage *j* to the future population. The primary reproductive curve spikes for the mites reared on the endophyte-colonized plants were lower than for those grown on the endophyte-free plants.

**Figure 7:**
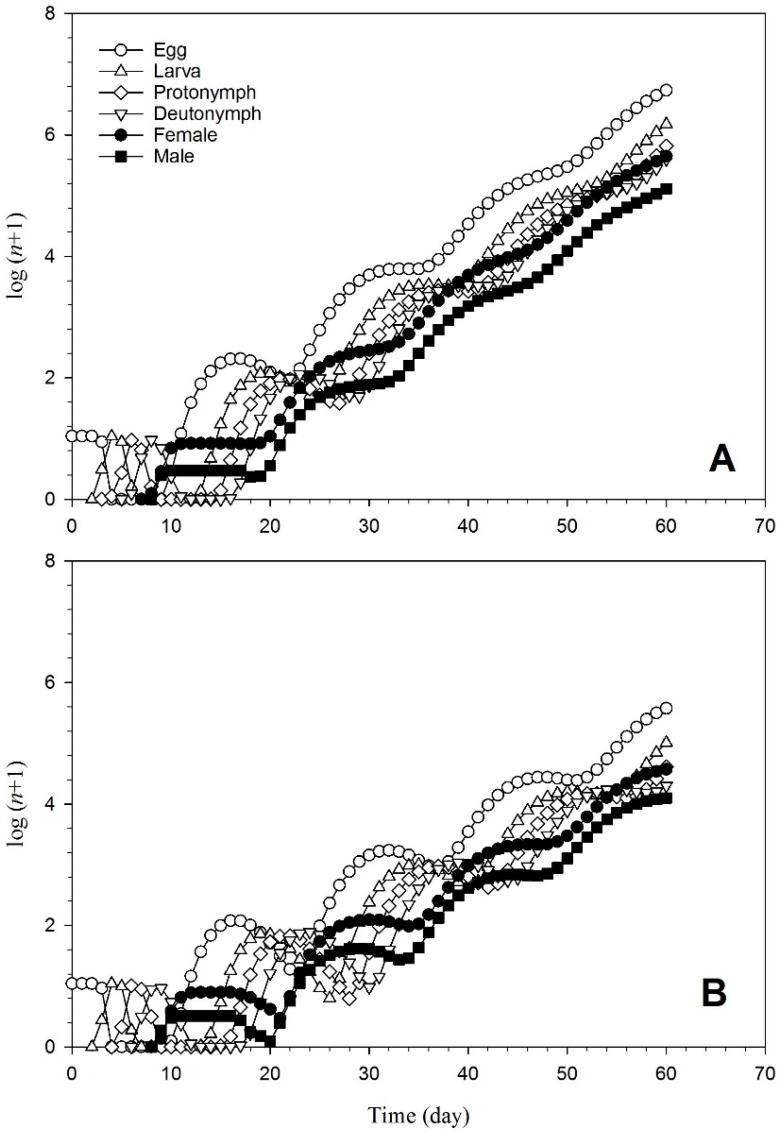
Population projection of *T. urticae* reared on endophyte-free (**A**) and colonized (**B**) plants.

*T. urticae* raised on the endophyte-colonized and the endophyte-free plants exhibit a population increase and stage structure, as demonstrated in Figure 7. The population grew far more slowly once 10 eggs were laid on the endophyte-colonized plants than the population reared on the endophyte-free plants. More generations were projected in the untreated plants for 60 days than in the treated plants.

## 4. Discussion

This paper addresses the management of spider mite issues, focusing on challenges like the development of resistance to traditional control methods. Using entomopathogenic fungal endophytes to reduce herbivorous pests is a novel approach that is increasingly tested in laboratory-based studies [26]. This approach has the potential to become a pest management tool. According to increasing research, entomopathogenic fungi may form symbiotic relationships with plant types called endophytes that safeguard a host plant from numerous abiotic and biotic stresses [27,28]. Herbivores often exhibit reduced growth, development, and reproduction when exposed to endophyte-colonized plants [29]. This is the first study to look at the life table of plants invaded by fungal endophytes in depth [9]. There has been some research on the sub-lethal effects of entomopathogenic fungi through the life table of *T. urticae* [2,30]; this study investigated the demography of *T. urticae* (Tetranychidae) on *P. vulgaris* (Fabaceae) plants colonized by entomopathogenic fungal endophytes to project the population dynamics of the mites under the influence of these endophytes [31].

In elucidating the intricate dynamics of *P. vulgaris* endophytic colonization by *B. bassiana* and its consequential impact on *T. urticae*, this study unveils compelling insights into the complex interplay between a plant, pest, and entomopathogenic fungus. Our findings showcase the successful establishment of *B. bassiana* across various plant tissues, echoing prior research on its efficacy as an endophyte [32]. The nuanced effects observed in *T. urticae*’s life history parameters and population dynamics contribute to a growing body of knowledge on the potential of *B. bassiana* in shaping ecological balance in agricultural ecosystems. Drawing parallels with the established literature, this study enhances our understanding of endophyte–pest interactions, offering a foundation for sustainable pest management strategies that harness the ecological roles of entomopathogenic fungi [33].

The investigation into *P. vulgaris* endophytic colonization by *B. bassiana* following inoculation unveiled an establishment of the fungal endophyte across various plant tissues, including leaves, stems, and roots [34]. The microscopic examination meticulously confirmed the presence of *B.* bassiana, revealing intricate hyphal structures and widely distributed conidia [35]. This observation aligns seamlessly with the findings of Wang et al. [36], underscoring *B. bassiana’s* remarkable efficacy as an endophyte in permeating diverse plant tissues, a phenomenon critical for its potential biocontrol applications [37].

A comprehensive exploration of the key life history data of *T. urticae* exposed to endophyte-colonized plants unveiled intriguing variations in developmental durations, particularly in the protonymph and deutonymph stages [38]. Notably, the cumulative pre-adult stage duration was significantly prolonged for the colonized plants, signifying a substantial impact on the overall population dynamics. These results resonate with the work of Zhu et al. [18], who reported that entomopathogenic fungi can exert notable effects on developmental parameters and longevity in arthropod pests.

Delving deeper into demographic parameters, such as the net reproductive rate (*R*_0_), the intrinsic rate of increase (*r*), and the finite rate of increase (*λ*), it became evident that endophyte colonization significantly influenced *T. urticae* population growth. The observed reduction in *R*_0_, coupled with alterations in the intrinsic rates of increase and finite rates, echoes findings from Birch [39], providing a compelling argument for the impact of endophytes on the reproductive potential of herbivorous pests.

The survival rate curves further illuminated the nuanced effects of endophyte colonization, particularly on female adults. The discernible implications for population dynamics echo prior research, which elucidated the role of endophytes in shaping age-stage survival curves and influencing population structure. These findings underscore the intricate interplay between endophytes and herbivorous pests, adding depth to our understanding of the complex ecological dynamics at play.

Fecundity and age-specific fecundity data added another layer to the narrative, revealing reduced fertility in endophyte-colonized plants [40]. This aligns harmoniously with investigations in which endophytes were shown to exert discernible effects on pest reproductive behavior. The detailed exploration of these facets in the current study contributes not only to the growing body of evidence surrounding endophyte–pest interactions but also provides nuanced insights into their potential implications for pest management strategies in agricultural systems.

The comprehensive examination of *P. vulgaris*’ endophytic colonization by *B. bassiana* and its cascading effects on the life history traits and population dynamics of *T. urticae* expands upon existing research and refines our understanding of these intricate ecological interactions. By drawing parallels with and extending findings from previous studies, this research offers a more nuanced perspective on the multifaceted relationships between endophytes and herbivorous pests, paving the way for informed and sustainable pest management practices in agriculture.

## 5. Conclusions

Finally, this study investigated the complex connection between the two-spotted spider mite *T. urticae* and the fungal endophyte *B. bassiana* in the setting of typical bean plants. We acquired beneficial knowledge about the changing patterns of mite populations raised on untreated and endophyte-colonized plants by employing the age-stage, two-sex life table hypothesis. Our research highlighted several significant differences in mite development and reproductive outcomes between the two plant conditions. The mites reared on the untreated plants exhibited shorter developmental stages and higher fecundity, resulting in a notably greater net reproductive rate and intrinsic rate of increase. Conversely, colonization by *B. bassiana* adversely affected multiple aspects of the mite’s life cycle, resulting in a reduced adult lifespan, a lower fecundity, and a diminished net reproductive rate and intrinsic rate of increase. These findings underscore the potential of entomopathogenic fungal endophytes, such as *B. bassiana*, in shaping integrated pest management strategies. Our research suggests that these endophytes may hold promise in host plant resistance programs, offering a natural and sustainable means to mitigate the impact of agricultural pests like *T. urticae*. By better understanding the complex interplay between mites, host plants, and beneficial endophytes, we can pave the way for more effective and eco-friendly pest control practices in agricultural systems. Further exploration of this area may yield innovative solutions to enhance crop resilience and reduce the need for chemical interventions in pest management.

## Figures and Tables

**Table 1:** Means (±SE) of the developmental durations, reproductive period, fecundity, and adult longevity of *T. urticae* exposed to untreated and fungal-endophyte-colonized bean plants.

Statistics	Untreated Plants (50)	Colonized Plants (50)
Development duration (d)		
Egg	3.78 ± 0.04 a	3.82 ± 0.04 a
Larva	2.08 ± 0.04 a	2.15 ± 0.03 a
Protonymph	1.76 ± 0.04 b	2.02 ± 0.06 a
Deutonymph	2.14 ± 0.04 b	2.45 ± 0.06 a
Total pre-adult stage	9.77 ± 0.08 b	10.49 ± 0.10 a
Preadult survival rate (*sa*)	0.93 ± 0.001 a	0.91 ± 0.001 a
Adult longevity (d)	12.88 ± 0.25 a	9.05 ± 0.13 b
APOP (d)	0.84 ± 0.001 a	0.81 ± 0.001 a
TPOP (d)	11.56 ± 0.13 a	10.76 ± 0.11 b
Total life span (d)	22.65 ± 0.27 a	19.54 ± 0.19 b
Fecundity (*F*) (eggs)	57.67 ± 1.45 a	28.01 ± 0.73 b
Oviposition days (d)	11.10 ± 0.23 a	7.50 ± 0.19 b

APOP = adult pre-oviposition period; TPOP = total pre-oviposition period. The bootstrap method was used to calculate standard errors with 100,000 resamples. The variance between the two groups was compared using the paired bootstrap test (TWOSEX-MSChart) between endophyte-free and colonized plants. Significant differences between the sexes are shown by means denoted by a different letter (*p* ≤ 0.05).

**Table 2:** Demographic parameters (mean ± SE) of *T. urticae* exposed to untreated and fungal-endophyte-colonized bean plants.

Parameters	Untreated Plants (50)	Colonized Plants (50)
Intrinsic rate of increase, *r* (d^−1^)	0.245 ± 0.005 a	0.196 ± 0.005 b
Finite rate of increase, *λ* (d^−1^)	1.27 ± 0.006 a	1.21 ± 0.007 b
Net fertility rate, *R*_0_	42.53 ± 3.03 a	19.26 ± 1.54 b
Mean generation time, *T* (d)	15.27 ± 0.12 a	15.04 ± 0.14 b

Means (SE) in the same row indicate major variations across treatments, and various letters follow. (paired bootstrap test, B = 100,000, TWOSEX-MSChart, *p* ≤ 0.05).

## Data Availability

The data sheets will be available upon reasonable request from the associated author.

## References

[1] Esmaeel R.E., Basha A., Mostafa E., Abd El Mageed A. (2018). Seasonal abundance of the two spotted spider mite, *Tetranychus urticae* koch on four cotton cultivars at Dakahlia Governorate, Egypt. Zagazig J. Agric. Res..

[2] Dash C.K., Bamisile B.S., Keppanan R., Qasim M., Lin Y., Islam S.U., Hussain M., Wang L. (2018). Endophytic entomopathogenic fungi enhance the growth of *Phaseolus vulgaris* L.(Fabaceae) and negatively affect the development and reproduction of *Tetranychus urticae* Koch (Acari: Tetranychidae). Microb. Pathog..

[3] Migeon A. (2007). Spider Mites Web: A Comprehensive Database for the Tetranychidae. http://www.montpellier.inra.fr./CBGP/Spmweb/.

[4] Boyle S.M., Salom S., Schultz P., Lopez L., Weber D.C., Kuhar T.P. (2023). Augmentative biological control for squash bug (Hemiptera: Coreidae) using the egg parasitoid, *Hadronotus pennsylvanicus* (Hymenoptera: Scelionidae). Environ. Entomol..

[5] Ding J.-L., Lin H.-Y., Hou J., Feng M.-G., Ying S.-H. (2023). The entomopathogenic fungus *Beauveria bassiana* employs autophagy as a persistence and recovery mechanism during conidial dormancy. mBio.

[6] Jaber L.R., Ownley B.H. (2018). Can we use entomopathogenic fungi as endophytes for dual biological control of insect pests and plant pathogens?. Biol. Control.

[7] Jaronski S.T. (2023). Mass production of entomopathogenic fungi—State of the art. Mass Prod. Benef. Org..

[8] Shahid A.A., Rao Q.A., Bakhsh A., Husnain T. (2012). Entomopathogenic fungi as biological controllers: New insights into their virulence and pathogenicity. Arch. Biol. Sci..

[9] Bamisile B.S., Siddiqui J.A., Akutse K.S., Ramos Aguila L.C., Xu Y. (2021). General limitations to endophytic entomopathogenic fungi use as plant growth promoters, pests and pathogens biocontrol agents. Plants.

[10] Van Loon L. (2007). Plant responses to plant growth-promoting rhizobacteria. New Perspect. Approaches Plant Growth-Promot. Rhizobacteria Res..

[11] Glick B.R. (2012). Plant growth-promoting bacteria: Mechanisms and applications. Scientifica.

[12] Thambugala K.M., Daranagama D.A., Phillips A.J., Kannangara S.D., Promputtha I. (2020). Fungi vs. fungi in biocontrol: An overview of fungal antagonists applied against fungal plant pathogens. Front. Cell. Infect. Microbiol..

[13] Beneduzi A., Ambrosini A., Passaglia L.M. (2012). Plant growth-promoting rhizobacteria (PGPR): Their potential as antagonists and biocontrol agents. Genet. Mol. Biol..

[14] Cocking E.C. (2003). Endophytic colonization of plant roots by nitrogen-fixing bacteria. Plant Soil.

[15] Branine M., Bazzicalupo A., Branco S. (2019). Biology and applications of endophytic insect-pathogenic fungi. PLoS Pathog..

[16] Gurulingappa P., Sword G.A., Murdoch G., McGee P.A. (2010). Colonization of crop plants by fungal entomopathogens and their effects on two insect pests when in planta. Biol. Control.

[17] Canassa F., Tall S., Moral R.A., de Lara I.A., Delalibera I., Meyling N.V. (2019). Effects of bean seed treatment by the entomopathogenic fungi *Metarhizium robertsii* and *Beauveria bassiana* on plant growth, spider mite populations and behavior of predatory mites. Biol. Control.

[18] Zhu H., Fu J., Wang H., Bidochka M.J., Duan M., Xu W., Sui L., Ren B., Li Q., Zhang Z. (2023). Fitness consequences of oviposition choice by an herbivorous insect on a host plant colonized by an endophytic entomopathogenic fungus. J. Pest Sci..

[19] Tuan S.J., Lee C.C., Chi H. (2014). Population and damage projection of *Spodoptera litura* (F.) on peanuts (*Arachis hypogaea* L.) under different conditions using the age-stage, two-sex life table. Pest Manag. Sci..

[20] Humber R.A. (1997). Fungi: Identification. Manual of Techniques in Insect Pathology.

[21] Chi H. (1988). Life-table analysis incorporating both sexes and variable development rates among individuals. Environ. Entomol..

[22] Chi H., Liu H. (1985). Two new methods for the study of insect population ecology. Bull. Inst. Zool. Acad. Sin.

[23] Chi H., You M., Atlıhan R., Smith C.L., Kavousi A., Özgökçe M.S., Güncan A., Tuan S.-J., Fu J.-W., Xu Y.-Y. (2020). Age-Stage, two-sex life table: An introduction to theory, data analysis, and application. Entomol. Gen..

[24] Hanife G., Saran C. (2021). Age-Stage, Two-Sex Life Table of The Diamondback Moth, *Plutella xylostella* (Linnaeus, 1758) (Lepidoptera: Plutellidae) on Different Brassicaeous Plants. Türk Tarım Ve Doğa Bilim. Derg..

[25] Kirby K.N., Gerlanc D. (2013). BootES: An R package for bootstrap confidence intervals on effect sizes. Behav. Res. Methods.

[26] Rosana A.R.R., Pokorny S., Klutsch J.G., Ibarra-Romero C., Sanichar R., Engelhardt D., van Belkum M.J., Erbilgin N., Bohlmann J., Carroll A.L. (2021). Selection of entomopathogenic fungus *Beauveria bassiana* (Deuteromycotina: Hyphomycetes) for the biocontrol of *Dendroctonus ponderosae* (Coleoptera: Curculionidae, Scolytinae) in Western Canada. Appl. Microbiol. Biotechnol..

[27] Gull A., Lone A.A., Wani N.U.I. (2019). Biotic and Abiotic Stresses in Plants.

[28] Subbarayalu Mohankumar S.M., Natarajan Balakrishnan N.B., Ramasamy Samiyappan R.S. (2012). Biotechnological and molecular approaches in the management of pests and diseases of crop plants. Integrated Pest Management: Principles and Practice.

[29] Al Khoury C. (2021). Can colonization by an endophytic fungus transform a plant into a challenging host for insect herbivores?. Fungal Biol..

[30] White N., Bale J.S., Hayward S.A. (2018). Life-history changes in the cold tolerance of the two-spot spider mite Tetranychus urticae: Applications in pest control and establishment risk assessment. Physiol. Entomol..

[31] Macuphe N. (2020). The Effect of the Entomopathogenic Fungus *Beauveria bassiana* on Growth, Physiological Responses and Control of Aphid (*Myzus persicae*) Infestation on *Lactuca sativa* L.. Ph.D. Thesis.

[32] Akello J., Dubois T., Gold C.S., Coyne D., Nakavuma J., Paparu P. (2007). *Beauveria bassiana* (Balsamo) Vuillemin as an endophyte in tissue culture banana (*Musa* spp.). J. Invertebr. Pathol..

[33] Athman S.Y. (2006). Host-Endophyte-Pest Interactions of Endophytic *Fusarium oxysporum* Antagonistic to *Radopholus similis* in Banana (*Musa* spp.). Ph.D. Thesis.

[34] Petrini O. (1991). Fungal endophytes of tree leaves. Microbial Ecology of Leaves.

[35] Riquelme M., Aguirre J., Bartnicki-García S., Braus G.H., Feldbrügge M., Fleig U., Hansberg W., Herrera-Estrella A., Kämper J., Kück U. (2018). Fungal morphogenesis, from the polarized growth of hyphae to complex reproduction and infection structures. Microbiol. Mol. Biol. Rev..

[36] Wang Z., Li M., Ju W., Ye W., Xue L., Boufford D.E., Gao X., Yue B., Liu Y., Pierce N.E. (2020). The entomophagous caterpillar fungus *Ophiocordyceps sinensis* is consumed by its lepidopteran host as a plant endophyte. Fungal Ecol..

[37] Torres-Rodriguez J.A., Reyes-Pérez J.J., Quiñones-Aguilar E.E., Hernandez-Montiel L.G. (2022). Actinomycete potential as biocontrol agent of phytopathogenic fungi: Mechanisms, source, and applications. Plants.

[38] Grabka R., d’Entremont T.W., Adams S.J., Walker A.K., Tanney J.B., Abbasi P.A., Ali S. (2022). Fungal endophytes and their role in agricultural plant protection against pests and pathogens. Plants.

[39] Birch L. (1948). The intrinsic rate of natural increase of an insect population. J. Anim. Ecol..

[40] Medeiros R., Ramalho F., Lemos W., Zanuncio J. (2000). Age-dependent fecundity and life-fertility tables for *Podisus nigrispinus* (Dallas) (Het., Pentatomidae). J. Appl. Entomol..

